# *Enskilment*: an Ecological-Anthropological Worldview of Skill, Learning and Education in Sport

**DOI:** 10.1186/s40798-021-00326-6

**Published:** 2021-05-21

**Authors:** Carl T. Woods, James Rudd, Rob Gray, Keith Davids

**Affiliations:** 1 Institute for Health and Sport, Victoria University, Melbourne, Australia; 2 Research Institute for Sport and Exercise Sciences, Liverpool John Moores University, Liverpool, UK; 3 Human Systems Engineering, Arizona State University, Santa Catalina Hall, Mesa, USA; 4 Sport & Human Performance Research Group, Sheffield Hallam University, Sheffield, UK

**Keywords:** Social anthropology, Ecological psychology, Ecological dynamics, Knowledge, Self-regulation

## Abstract

The aim of this paper is to explore a different, more relational worldview of skill, learning and education in sport. To do this, we turn to the work of social anthropologist, Tim Ingold, leaning on the notion of enskilment, which proposes that *learning* is inseparable from *doing* and *place*. From this worldview, what is learned is not an established body of knowledge, transmitted into the mind of a passive recipient from an authorised being, but is a progressively deepening embodied-embedded attentiveness, where an individual learns to self-regulate by becoming more responsive to people and environmental features by ‘looking, listening and feeling’. As we discuss, Ingold’s perspectives on enskilment are rooted in the etymological connotations of education—*ex-ducere*, which roughly means ‘to lead out’. In applying this notion to sport, we unpack three of its entangled components, *taskscapes*, *guided attention*, and *wayfinding*, detailing the implications of each for the growth of *enskilled* sports performers. To promote the translation of these ideas, in addition to encouraging their inquiry beyond the scope of what is discussed here, sporting examples are threaded throughout the paper.

## Key Points

- Theoretically situated within an ecological-anthropological framework, this paper explores a relational, transdisciplinary worldview of skill, learning and education in sport.
- We introduce the notion of *enskilment* to the sport sciences: a concept that proposes learning is inseparable from doing and/in place, meaning individuals become more actively self-regulating in performance through deepening their attentiveness to environmental features.
- In applying the notion of enskilment to sport, we thread through its key, entangled components, *taskscapes*, *guided attention* and *wayfinding*, conceptualising and exemplifying each within sport.

## Introduction

There is an insightful excerpt at the start of Tim Ingold’s book, *The Perception of the Environment* [1], in which he elaborates on the way his father taught him botany:

“When I was a child my father, who is a botanist, used to take me for walks in the country-side, pointing out on the way all the plants and fungi – especially the fungi – that grew here and there. Sometimes he would get me to smell them, or to try out their distinctive tastes. His manner of teaching was to show me things, literally to point them out. If I would but notice the things to which he directed my attention, and recognise the sights, smells and tastes that he wanted me to experience because they were so dear to him, then I would discover for myself much of what he already knew.” (p. 20)

As is apparent in this quote, knowingly or not, Ingold’s father seems to have adopted a profound pedagogical approach in advising him of botany. He encouraged Ingold to actively and directly experience the sights, smells and tastes of things, interacting with them in a way that allowed him to discover things for himself, yet in a safe and supported manner. To quote Reed ([2], p. 54), it appears that Ingold’s father viewed teaching “in the sense of helping others to learn the affordances of objects and tools within the context of a given skill”. Moreover, his approach seemed to be grounded in the etymological roots of education—*ex-ducere*, which roughly means ‘to lead out’ [3]. This embedded and embodied approach was intended to fine-tune Ingold’s senses toward the perception of things in the environment he was being led out into. Simply, Ingold was learning to become *attentive*^1^ to things by (self)discovering and actively engaging with them, all while under the careful guidance of his experienced father.

In musing over this excerpt, we were drawn into considering what education, learning and skill have come to mean in the sporting landscape, leading us toward two paths. One, situated in a Cartesian worldview, adopts an internalised unit of analysis, implicating the idea that to educate is to impart or transmit knowledge, by way of rules, schema or programmes, into the mind of a recipient, who then decodes them to be enacted by the body in a certain environmental context (e.g. [4, 5]). The other, situated in an ecological approach, sets its unit of analysis at the level of organism-environment interactions, implicating the idea that to educate (*ex-ducere*) is to guide the attention of individuals toward the perception of environmental features that could be used to support and (self)regulate emergent, adaptive behaviours (e.g. [6, 7]).

The latter is a path that Ingold’s father literally took him along, and years later, is what he referred to as an enskilment approach^2^ when explaining how people become skilful at activities in various places [1, 8]. Here, we elaborate on this social anthropological concept to understand (en)skilfulness in sport, guided by the contemporary theoretical framework of ecological dynamics [9]. In doing so, we explore three of its entangled components, *taskscapes*, *guided attention* and *wayfinding* [10], detailing what each may mean for the growth of enskilled sports performers. However, before setting off along this path and attending to its features as we go, we briefly detour down the former, and perhaps more traditional path, drawing our readers’ attention toward some fundamental *differences* in worldview, contrasted with ecological propositions on skill, learning and education.

## A Traditional Worldview of Motor Skill

Traditionally, motor skill learning has focused its unit of analysis almost exclusively on the organism (the individual or performer, for example), drawing metaphoric comparisons to computational processes putatively occurring in the mind to explain relatively permanent changes in movement capabilities [11]. This assumption is grounded in the notion that the brain functions like a computer to process inputted information, construct or calculate a response and then output this response in some behavioural form [12, 13]. The explanatory rationale for this computational perspective suggests that the environment is *impoverished*, meaning that information residing in it is not of sufficient quality, on its own, to control action, but merely offers *cues*. These cues are then integrated and processed by the brain to construct representations that are then purported to support outputted action [14]. These processes for perceiving and acting are, thus, dependent on internalised representations which are symbolic, indirectly helping the organism ‘know’ about the world through the consolidation of procedural and propositional rules that specify how a movement is to be controlled and organised relative to certain environmental features (i.e. objects or surfaces) [15]. What is ‘acquired’ in skill acquisition, then, is a set of internalised rules or propositional commands that specify how movements are to be carried out and controlled by the body.

This traditional worldview clearly impacts how practitioners would go about educating individuals to ‘acquire’ a skill. For example, if a skill is viewed as the enactment of an internally stored thing that specifies how movement sequences should be controlled, education would be conceived as a top down process of *transmission* [8]. Practitioners need to then attempt to impart (or transmit) knowledge into the minds of novices by way of repetitive and isolated practice and rehearsal of movement components or sequences, coupled with consistent and corrective instruction and feedback to ensure that the internalised control commands are ‘correct’ [16]. In sport, the complexity of coordination patterns shapes the deconstruction and decontextualisation of actions in order to master components separately before being put back together to be applied in context (e.g. breaking up a tennis serving action into distinct phases to be rehearsed and mastered in isolation before being progressively put back together to make up the full service action). Teaching someone to become skilful, then, would be an indirect process [17] of ‘filling up’ their stored knowledge about the task and environment to ‘instil’ idealised ways of ‘being and doing’ that fit established conventional templates [1, 8]. As we now go on to show, these ideas are radically different to those that reside within an ecological rationale, and it is here that we negotiate, with our readers, a *different*, more relational path to explain movement skill, coordination, learning and education in sport.

## Toward a *Different* Worldview of Motor Skill Learning

Given the tenets of a traditional worldview of motor skill learning within cognitive science [13], there are growing calls to restore balance between the organism and environment when explaining skilled behaviours, such as coordination and its acquisition (e.g. [18–20]). These calls, grounded in a theory of direct perception and action in ecological psychology [7, 21], fundamentally refute the idea that skill and learning can be explained by ways of indirect symbolic representations of the world and computational processes occurring in the mind. Rather, skill ‘acquisition’ is viewed as pertaining to a dynamic and evolving *fit* between the action capabilities of an organism, the task to be achieved, and the environmental niche which they inhabit [19]. So, rather than an entity being acquired, an evolving and functionally adaptable fit *emerges* between an organism and the constraints of his/her environment as they progressively attune to information that specifies opportunities for (inter)action [7]. These ideas necessarily focus attention on coordination of actions, continuously regulated by the information that surrounds us, leading to a more functional relationship with a performance environment [22]. Over many decades, they have grown in traction within global organisations and institutions of the sporting landscape, captured in the contemporary lens of ecological dynamics (for regular updates of key concepts and empirical evidence see [23–25]).

From this worldview, education is not a process of knowledge transmission informed about how something ‘should’ be done, but is a process in which an individual is guided along a path of self-discovery [26]. While on this path, individuals are encouraged to directly experience things for themselves, meaning knowledge is not an entity to be transmitted into the receptacle mind of an individual, but is ‘grown’ as they actively find their own way through various performance landscapes, while continuously guided, mentored and supported by an experienced other [1, 3, 8]. As captured by Ingold ([8], p. ix], this educative process “is about attending to things, rather than acquiring the knowledge that absolves us of the need to do so; about exposure rather than immunisation”. Learning, then, is not a process of *enculturation* (manifested in rules, symbols or representations about how things should be done relative to convention or template), but is one of *enskilment* [1]. During enskilment, individuals are encouraged to experience the sights, sounds, feelings and smells of things, attending to these sources of information directly as they are, embedded into the context in which they exist. To elaborate on these points, we now progress with our readers down the path that is enskilment.

## Enskilment

We start our journey with a key idea from Ingold ([1], p. 416), which highlights the embodied-embedded undertones of what it means to be enskilled^3^:

“‘Understanding in practice’, by contrast, is a process of enskilment, in which learning is inseparable from doing, and in which both are embedded in the context of a practical engagement in the world.”

This conceptualisation emphasises that enskilment can be viewed as a deep, tacit and practical knowledge—a type of local ‘know how’ or ‘knack’ that progressively emerges as an individual becomes intimately familiar with a task and surrounding. There is an important point to briefly highlight here, though, which is that this knowledge should not be confused as being proceduralised, manifested via the storage of prescriptive rules that are presumed to enable the automatisation of movement. Rather, from an enskilment perspective, knowledge is understood as a progressive attunement of one’s perceptual system toward the detection of information in the environment of use for regulating action. This is knowledge that is grown by ‘doing’ and actively engaging with one’s environment [27], and is why Ingold [1] regularly refers to enskilment as being the continued process of *attending* or *responding* to things; deeply aligned with the key ecological concept of the *education of attention* [7], which emphasises the importance of helping individuals find the most relevant sources of regulatory information during interactions with a performance environment.

This view of enskilment immediately cuts through the division of mind and body and body and world that a traditional worldview of motor skill learning creates, a sentiment emphasised by Pálsson [28 p. 904], stating that (with an enskilment approach) “the proper unit of analysis is no longer the autonomous individual separated from the social world by the surface of the body, a natural being who passively internalises the mental scripts of the cultural environment, but rather the whole person in action, acting within the contexts of that activity”. Thus, becoming enskilled cannot occur separately, in isolation from context or experience, as it grows in the messiness of the noisy ‘real-world’. This notion, again, aligns with the contemporary framework of ecological dynamics, viewing skilful actions not as repetitions of movements and sequences detached from context, but as dynamic, body-environment interactions that individuals learn to actively self-regulate by perceiving opportunities for action in achieving intended task goals [29].

These place-based, relational ideas of enskilment are of critical importance in explaining skill, learning and education within the sporting landscape. Indeed, governing rules in sports can shape intentionality through the ‘legality of actions’ (like not being able to double dribble in basketball, or keeping one foot placed in contact with the ground during race walking). But there is a critical difference between knowing about the rules of a game, and possessing a finely tuned perception-action coupling that enables one to (en)skilfully ‘play’ the game [30]. For example:

- To successfully dribble the ball down field, a footballer would need to (among other things) *educate their attention to information about* the continued movements of defenders, teammates, the time of the game, the ground surface properties, and the movement of the ball;
- A cricket batter would need to *educate their attention to information about* the arm and wrist position of the bowler, the rotation of the ball in flight, its bounce off the surface of the wicket, and the position of fielders to successfully score runs;
- A golfer teeing off would need to *educate their attention to information about* the surface on which the ball is sitting, the line, layout and textures of the green (i.e., its hazards), and the wind direction and strength when attempting to make par.

The important thing to highlight here is that these sources of information are constantly and dynamically evolving (along with the action capabilities of the performer). This means that an enskilled sports performer cannot pre-programme actions but must be adaptive and responsive to the ever-changing environment, *submitting* to the unpredictability of the environment every time they head out to ‘play’—or, as eloquently summarised by Ingold ([31], p. 138):

“Thus the walker, a master of the terrain, must *wait* for signs that reveal the path ahead, with no surety of where it will lead; the hunter, a master of the chase, must *wait* for the animal to appear, only to put himself at risk in its pursuit; the mariner, a master of his ship, must *wait* for a fair wind, only to submit to the elements.” (emphasis added)

So, a highly experienced and enskilled footballer, cricketer or golfer would then not be an individual who can better represent the world, but one who is more attuned to specifying information that can be used to support the regulation of action toward the achievement of a task within their ecological niche (i.e., performance environment) [7]. That is, from an ecological dynamics rationale, highly skilled individuals have established (and continue to develop) a functionally adaptable fit between their evolving action capabilities and the affordances of the performance environment [19]. The role of a coach or experienced other is, then, not necessarily to solve problems for someone by ways of only prescriptive instruction or continual feedback. Rather, it is to help them grow an intimate *‘knowledge of’* [21] the performance landscape and its possibilities for action. An analogy that we find of use here is that of a map—a map may *initially* help orient an individual who is about to hike an unfamiliar region (like guiding instructions from a coach *about* how an individual may like to *start* exploring an unfamiliar task). But an enskilled hiker is more interested in supporting their journey by progressively learning about the terrain, flora and fauna, climate, celestial movements and local history (for example)—things difficult to directly experience and thus come to ‘know of’ by only ever following markings scribed onto the surface of a map!

**Fig. 1:**
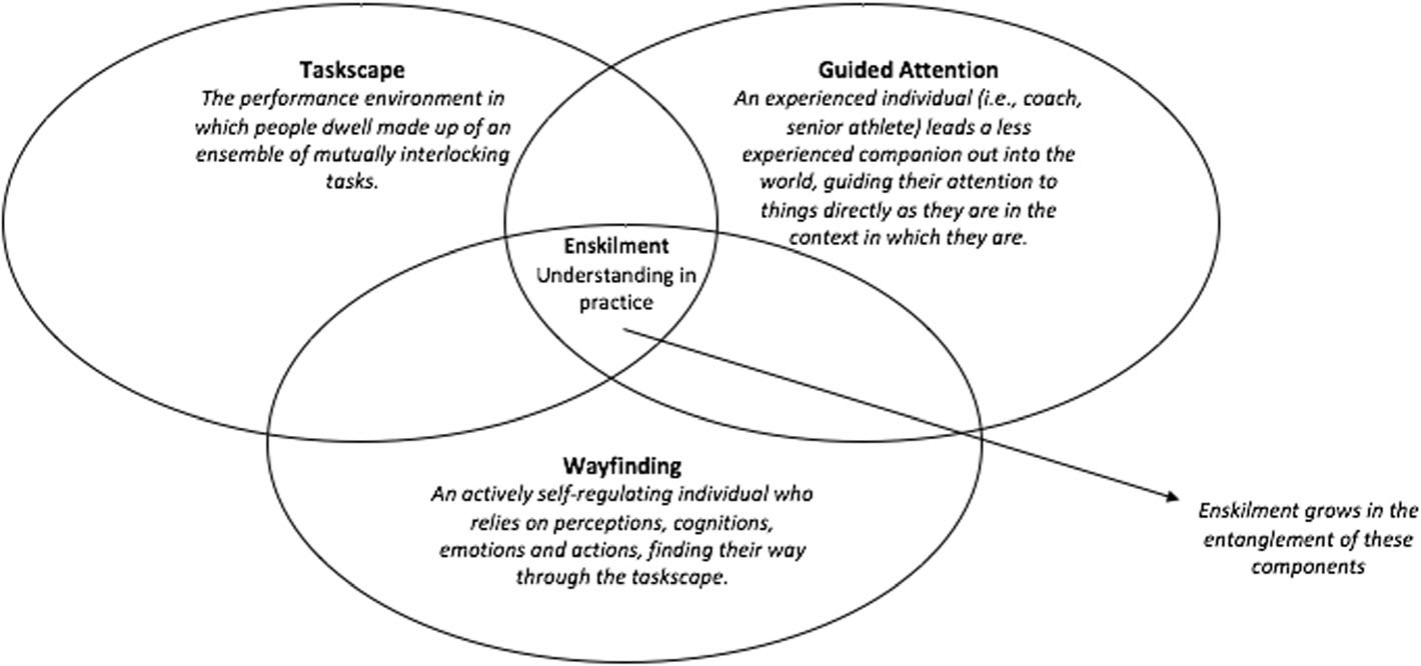
The growth of an enskilled sports performer. Each component is entangled to support enskilment—the taskscape: viewed as the performance environment; guided attention: viewed as the role of the practitioner; and wayfinding: viewed as an actively self-regulating performer

So, in coming to this point along our path, an apparent question arises: how would an experienced other (i.e., a coach or senior athlete) go about helping a less experienced other (i.e., a less experienced athlete) in becoming enskilled? More deeply, how does one support the growth of responsivity and attentiveness that is required to self-regulate perceptions, emotions, cognitions and actions when becoming enskilled in sport? In attending to these theoretical questions of practical relevance, we next explore three components of enskilment as highlighted by Prins and Wattchow [10]: *taskscapes*, *guided attention* and *wayfinding*. We conceptualise each within the sporting landscape, showing that it is in their entanglement where enskilment is grown—it is the *taskscape* in which an activity resides (what we conceptualise as the performance environment in a sport), *guided attention* that deepens one’s knowledge of the activity within the taskscape (what we conceptualise as being a key part of the role of the practitioner in sport), and *wayfinding* that promotes the growth of active self-regulation (what we conceptualise as the performer interacting with a *taskscape* (performance environment)) (Fig. 1).

### Taskscape: the Performance Environment

In building toward his description of the taskscape, Ingold [1] emphasises that people become enskilled by dwelling within various places—meaning that learning about a place is inseparable from being *in* a place. It is by dwelling that people learn to become familiar with a place’s features, which includes the tasks of other inhabitants that “make up the pattern of activity of a community” ([1], p. 325). From this dwelling perspective, a task can be understood as a practical, goal-directed activity undertaken by an individual interacting with an environment [1]. So, then, the *taskscape* is the “entire ensemble of tasks in their mutual interlocking” ([1], p. 195). It is important here to highlight the words ‘mutual interlocking’, as they emphasise that tasks are never completed in isolation, but only exist in an enmeshment of people interacting with each other and a performance environment. This conceptualisation points toward a certain rhythmicity, temporality and self-organisation of the taskscape, suggesting that, as people and environmental features change (over varying timescales), and so do the task dynamics. What this means for the enskilled performer, then, is that they must be sensitive and responsive to even the most subtle of changing rhythms, as it is this attunement to the information of a performance environment that likely preserves functionality and self-organising tendencies in the taskscape.

When the idea of a taskscape is applied to sport, it becomes apparent that learning is more than simply reproducing some technique or movement template removed from context. Downhill mountain biking, for example, would require an individual to perform a variety of different, but interlocking, tasks if they are to become an enskilled rider. To exemplify, they would need to learn to attend to the trails, looking out for varying ground surface properties (like loose pebbles or overly soft dirt) or obstacles (like trees, shrubs, holes or large rocks) that all offer different opportunities, some of which could be potentially hazardous, cause them to fall, or simply slow their speed in a time trial. Riders would need to learn to be attentive to the varying weather conditions, learning how recent rain, wind or dry weather could alter course properties, implicating the routes chosen. It may also require them to learn to fix a punctured tire, snapped chain or bent tire spoke, tasks especially critical if riding along remote tracks, and perhaps without a full suite of specialised tools. It could involve learning about social rules and norms while on the track by interacting with experienced riders who also dwell within the performance environment. Progressing further, the rider may even learn to customise features of their bike such that they better align with specific body dimensions and/or local track characteristics, feeling more ‘in tune’ with the rhythms of track when riding in a familiar place. The key point is that from an enskilment perspective, sporting tasks, like downhill mounting bike riding, cannot be viewed as an isolated action, but rather as an emergent, embedded and embodied task that is influenced (and influences) a broader array of interlocking tasks that function to make up the taskscape. Simply, what an enskilled sports performer learns is not an idealised way of doing, but how a task functionally *fits* within the broader rhythms of the taskscape. Viewed in such a way, an enskilled downhill mountain biker would enter into a ‘poetic involvement’ with the taskscape [1], learning to resonate with, and attend to, the dynamic information on sights (e.g. track surface properties), sounds (e.g. discussions between fellow riders) and feelings (e.g. of bike on different ground surfaces) that progressively emerge.

From an ecological dynamics rationale, these ideas align nicely with those of representative learning design [32]. Founded on the initial ideas of *representative design* by Brunswik [33], representative learning design indicates that practice environments should be ‘representative’ of the key informational constraints that sports performers experience during competition [32]. Representative learning design requires that practice contexts simulate performance environments, preserving the coupling between perception and action, helping a sports performer to continue to establish a deep and evolving fit with their competitive environment [19, 34]. Thus, having established that the taskscape is *where* and *what* we learn, our focus now shifts to that of an experienced other (i.e., coach or senior athlete) and the role they play in the growth of an enskilled sports performer.

### Guided Attention: the Role of the Experienced Practitioner

To this point, our readers could be excused for thinking that, within an enskilment approach, the role of an educator is minimal. This view, however, would be a misinterpretation likely founded on the traditional meaning of education—that is to impart knowledge into someone via rules or defined ways of doing [3]. From an enskilment perspective, though, education is conceptualised in its etymological roots—that of *ex-ducere*, meaning to lead someone out into the world [3]. Thus, far from being a minor part of the learning process, the experienced practitioner is an integral part of the taskscape, walking *with* the less experienced other along paths of inquiry that open as they go, or as Ingold ([8], p. ix) states:

“The task of the educator, then, is not to explicate knowledge for the benefit of those who are assumed, by default, to be ignorant, but to provide inspiration, guidance and criticism in the exemplary pursuit of truth”

There are some fundamentally different aspects of the role of an educator, considered from an ecological dynamics perspective, compared to a more traditional understanding. It is an educative process that, in itself, requires careful enskilment. For example, as captured in the above quote, helping someone become responsive to subtle features of the taskscape requires inspiration, patience, support, creativity and a deep understanding of a companion’s action capabilities relative to the task they are engaged with. It can be viewed as a process of active self-discovery—‘active’ in the sense that it is a relational interaction between an experienced and less experienced other dwelling in a performance environment [29], but ‘self-discovery’ in the sense that the less experienced other is encouraged to attend to things directly as they are, in the context in which they are [1].

This approach to education, we argue, is ‘hands on’, not in the hard sense of instructing, but in the soft sense of *guiding*—guiding one’s attention toward the direct perception of things that may otherwise be cloaked to them by watching, listening or feeling [1, 7, 8]. At this point, as done with the roots of the word ‘education’, it is important to briefly emphasise the etymology of ‘attention’—*ad-tendere*, meaning to ‘stretch toward’ [8]. It is in this *stretching toward* where we actively connect with or relate to things, like actively *listening* to the sound of a teammate’s voice (in a confluence of other sounds) warning of an approaching defender in a football game, actively *watching* the movements of an opponent in a tennis game to exploit a subtly progressive weakness emergent in their game, or actively *feeling* the wind's strength and directional changes through a boat’s sails when tacking on the water.

Guiding a performer’s attention to informational properties that support performance in the taskscape can take shape in a variety of ways. For example, while engaging with an inexperienced cricket batter who is learning to become enskilled, a coach or senior teammate (perhaps even batting *with* them at the non-striker’s end) may use subtle gestures, like facial expressions or body movements to sample available informational constraints. These exploratory actions can guide their teammate’s attention toward the perception of affordances (e.g. the re-positioning of fielders, gaps appearing on-field, the position of the ball in the bowler’s hand, subtle changes in weather patterns like wind direction or ambient lighting, or cracks emerging in the pitch). The detection of information for the perception of available affordances could be used to support the decisions and actions taken, such as the type of shots played. Importantly, while these information sampling movements can be of use to support, challenge or reinforce behaviours within a certain context, they can also establish a deep rapport between an experienced and less experienced other. Such a rapport can help individuals learn to attend to (i.e., stretch toward) each other’s gestures in a type of responsiveness that is only grown by mutually dwelling within a particular performance environment [1].

This guidance for individuals may also take shape in the design of practice activities intended to channel one’s attention toward the perception of critical features of the taskscape [35]. This idea bodes particularly well with key concepts within an ecological dynamics framework. For example, sports practitioners functioning within this framework are often encouraged to view themselves through a ‘learning designer lens’ [25], working with athletes to place the individual-environment interaction at the core of the practice design [36]. This approach is intended to encourage, through co-design, the athlete to seek, discover and exploit available affordances realised in the completion of a task [36], growing their *knowledge of* [21] the performance environment. For example, an experienced football coach working with a youth team could design an activity that guides the attention of players toward the discovery and exploitation of ‘space’ by carefully manipulating features of the taskscape. Notably, they could reduce the number of defenders or alter the playing area dimensions in a practice activity to accentuate temporal and spatial features of taskscape during an attacking sub-phase of play. Or, they could anchor points/scores to actions that emerge during play to create space for teammates, using bodily gestures, such as clapping or hand signalling, highlighting examples for modelling, to help players connect to information that directly supports functional behaviours.

In sum, the role of the coach in an enskilment approach is predominantly one of guidance, and while at times this guidance may require slight nudging or even showing, it rarely starts and ends in the ‘hard pedagogical act’ of *instructing*, engrained in a specific way of doing. Guiding, nudging, modelling or showing are ‘softer pedagogical approaches’ that intend to unveil a world to be further explored and discovered by a performer, starting from varying vantage points. They are pedagogical actions, often undervalued, that are intended to open lines of inquiry to be followed up in future interactions by the performer in a kind of (active, self-regulated) self-discovery, followed by reflection. Hard pedagogical actions such as *instructing*, *informing* or *telling* (on what to do), in contrast, risk a fixation on a prescribed movement template, outcome or destination (i.e., an acquired way of doing). In fixating upon directing a performer towards a pre-defined, rigid destination (like strictly following *just* the markings scribed on a map or the steps in a recipe), a practitioner risks decoupling perception from action, limiting an individual’s capability to sharpen knowledge of the subtleties of the taskscape (by detaching person from place), dampening their capability to learn to actively self-regulate in performance: exploratory activities which define the very ecological commitments of learning and which sit at the very roots of what it means to be enskilled.

### Wayfinding: the Actively Self-Regulating Performer

Wayfinding is a narrative way of moving through a landscape [1]. It is narrative because, as moving through it, an individual progressively and actively learns about its surface properties, layout, climate, vegetation, history and socio-cultural norms through stories told, questions asked and experiences gained. Stories and questions are typically used by an experienced other to help a less experienced companion learn the rhythms of the taskscape in a more intimate way and are thus used while both are embedded into the taskscape [1]. From this perspective, wayfinding is far more than simply navigating *across* a landscape to reach some fixed point in space. This detached view is better understood as *transport*, where an individual relies on a map, compass or global positioning satellite to inform them of their location relative to a known destination [29]. Navigating is analogous to a procedural manual that may break a ‘skill’ up into sequential parts to be ‘mastered’ in isolation by a novice—with these parts being checked off relative to the criteria scribed onto an operating manual as to what these movement components should ‘look’ like, before being reverse-engineered as a whole. An enskilled wayfinder, by contrast, is generally not interested in ‘learning by numbers’, following a pre-specified route that leads to a terminus destination, but is more interested in attending to environmental features that emerge along a journey; a sentiment captured by Ingold ([1], p. 242):

“But above all, wayfinding depends upon the attunement of the traveller’s movements in response to the movements, in his or her surroundings, of other people, animals, the wind, celestial bodies, and so on. Where nothing moves there is nothing to which one can respond.”

This perspective on learning behaviour indicates that successful wayfinding requires a deep and practical knowledge of the environment that one inhabits, as it is this knowledge that supports an individual’s capacity to actively self-regulate through it by way of finely tuned perceptual and action systems [37]. Further to this, the more varied and dynamic the environment, the richer the opportunities an individual has to learn what to (and not to) attend to when finding their way. An enskilled and actively self-regulating wayfinder is, therefore, one who is attentive and responsive to even the most subtle rhythms and patterns that emerge in the taskscape. It is this attentiveness that keeps them ‘in touch’ with the world, even helping them find their way through uncharted terrain. Thus, enskilled wayfinders are rarely ‘lost’ or ‘alone’ when situated within the places they inhabit, as the environment itself provides relations that support interactions needed to continually regulate behaviour.

The idea of learning to negotiate and move through performance environments in sport has been espoused through the framework of ecological dynamics (e.g. [29]). We argued that, while typically not physically traversing through different regions of a landscape, sports performers could (metaphorically) be viewed as wayfinders, progressively learning to self-regulate in dynamic, complex performance environments by using perceptions, emotions, cognitions and actions to solve emergent problems within the taskscape [29]. We proposed that learning designs in sports need to provide performers, at all levels, with carefully graded and well-designed opportunities to develop relevant wayfinding behaviours to enrich their self-regulating capacities. For example, in helping a young tennis player learn to wayfind through the emergent problems of a competitive match, a coach could use carefully targeted questions that show the player where to look, but that do not prescribe what to see [38]. This pedagogical approach could take shape in carefully designed practice tasks. These tasks could be accompanied by relevant ‘open’ questions that challenge the young player to perceive information on: *where* an opponent is placed on the court, *how* this varying depth in court position could implicate an opponent’s capability to reach balls placed in specific locations on court, and *how* they could manipulate their racquet head to generate different types of spin on the ball to further accentuate competitive advantages afforded by an opponent’s court position. The point of such questions is not to prescribe answers that need to be verbalised by the player, as that would require *knowledge about* the environment. Rather, this type of questioning by the coach is a soft pedagogical act that helps the performer embedded in the taskscape to grow *knowledge of* it. The questioning could prompt the performer, when on court, competing against an opponent, to explore and discover information for self-regulation when exploiting available affordances for action. The aim of such questioning is to guide performers to attend to subtle features of the performance environment, available in surrounding information (i.e., optic, acoustic, haptic, proprioceptive), to support behaviour in situ (i.e., answering the question by acting, as opposed to verbalising). This type of pedagogical design emphasises wayfinding activities, helping to enskill performers to become more self-regulating in performance and less reliant on the augmented information in the prescriptions of the coach, parent or instructor.

## Conclusion

To conclude, we return to the insightful epigraph from Ingold’s book with which we started our paper. It is clear that Ingold’s father was indeed supporting him in becoming an enskilled botanist. Notably, the young Ingold was being led out into the *taskscape*, learning about its mutually interlocking features as his father *guided his attention* toward the tastes and smells of things, using such things to help him *wayfind* through a terrain that may have otherwise been unfamiliar and full of uncertainty. Such soft pedagogical actions emphasise that, from an enskilment perspective, skill, learning and education is (i) *inseparable from doing and place*, replete with contextual information; (ii) emergent somewhere in the *entanglement with the taskscape* (i.e., in sport situated as ‘the performance environment’); (iii) supported by *guided attention* (i.e., by parents, coaches or peers, such as a senior teammate); and (iv) viewed as *wayfinding* (i.e., the athlete or performer learning to solve problems and take decisive actions by attending to important things within the performance environment).

These perspectives point toward the very core of enskilment, which is founded on a relational, interactive way of being. It is a worldview that calls for humility, genuine inquiry and an embracement of the unknown. This approach accepts that at any time, an enskilled individual is both prepared and unprepared for the demands of the taskscape—prepared in that they are responsive or ‘tuned in’ to the opportunities for action, but unprepared in that they appreciate nothing is given in an environment that is constantly changing:

“To embark on any venture – whether it be to set out for a walk, to hunt an animal or to sail the seas – is to cast off into the stream of a world in becoming, with no knowing what will transpire.” ([31], p. 138)

This view advocates that no one, nor thing, should be seen as having all the answers, but that the answers emerge as people head out into the world together. So, although this paper is coming to an end, we encourage readers to progress on, continuing further down the path that is enskilment—perhaps using the ideas explored in this paper as a set of threads in which to respond and attend to—not to reach a ‘final’ destination, but to dwell, even briefly, within the various regions they encounter along the way.

## Data Availability

Not applicable

## Footnotes

1 Discussed in detail later, it is important that we briefly align our interpretation of ‘attention’. Following Gibsonian ideas, we view being ‘attentive’ as being *attuned* to information within one’s environment. Thus, the more attentive one becomes, the more attuned their perceptual system at ‘picking up’ subtle features of the environment of use to directly regulate action.

2 It is worth briefly expanding on the notion of enskilment here. It is a phrase used by Ingold [1] to argue that *learning* is inseparable from *doing* in *place*, in which people learn to become more attentive to various things within an environment they inhabit with others.

3 We wish to offer some brief context to this quote for readers less familiar with Ingold’s [1] text. This quote was used by Ingold to contrast with that of *enculturation* to argue that learning does not “entail an internalisation of collective representations or, in a word, *enculturation*” (page 416, emphasis in original). To Ingold, as indeed to us the authors of this current work, learning and skill emerge as people practically engage in activities with others, in place, progressively attending to things as they are, where they exist.
